# Supplementary material for: Collective unstructured interactions drive chromatin binding of transcription factors
Source: bioRxiv. 2025 May 23:2025.05.16.654615. Preprint. [Version 2] doi: 10.1101/2025.05.16.654615 (PMC12132478; doi:10.1101/2025.05.16.654615)
Supplement: 1 [file NIHPP2025.05.16.654615V2-supplement-1.pdf]

bioRxiv preprint doi: https://doi.org/10.1101/2025.05.16.654615; this version posted May 23, 2025. The copyright holder for this preprint (which was not certified by peer review) is the author/funder, who has granted bioRxiv a license to display the preprint in perpetuity. It is made available under aCC-BY 4.0 International license.

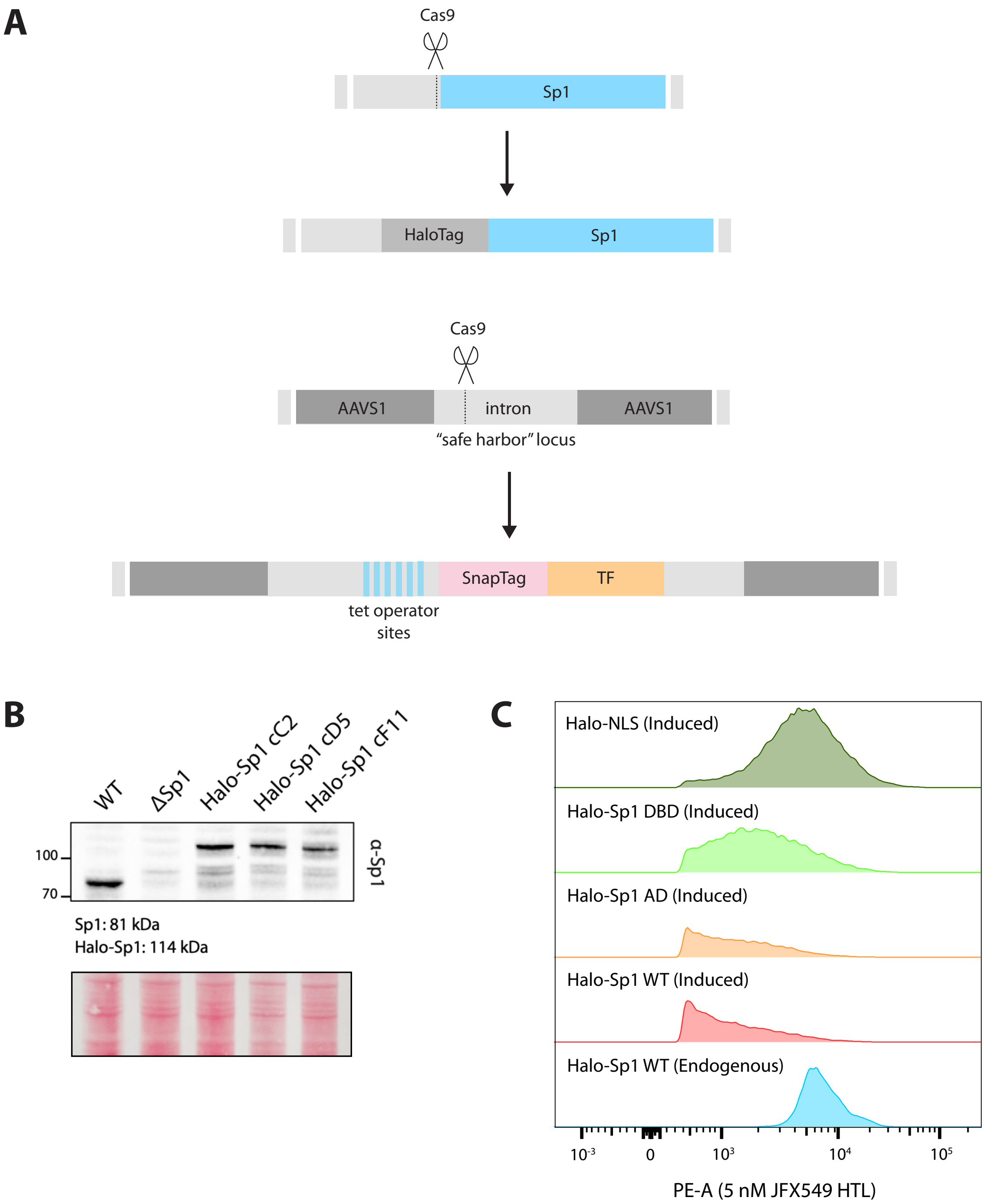

Figure S1. Engineering strategy for HaloTag and SNAPfTag fusions enables PAPA measurements at sub-physiological expression levels.
(A) CRISPR-Cas9 editing was used to engineer a HaloTag fusion at the *Sp1* endogenous locus. SNAPfTag fusion proteins driven by an inducible promoter were integrated at the *AAVS1* locus. **(B)** Western blot shows correct band size for HaloTag knock-in as well as Sp1 knockout. **(C)** Flow cytometry quantifies fluorescence for inducible transgene expression and endogenous HaloTag-Sp1 fusion.

bioRxiv preprint doi: https://doi.org/10.1101/2025.05.16.654615; this version posted May 23, 2025. The copyright holder for this preprint (which was not certified by peer review) is the author/funder, who has granted bioRxiv a license to display the preprint in perpetuity. It is made available under aCC-BY 4.0 International license.

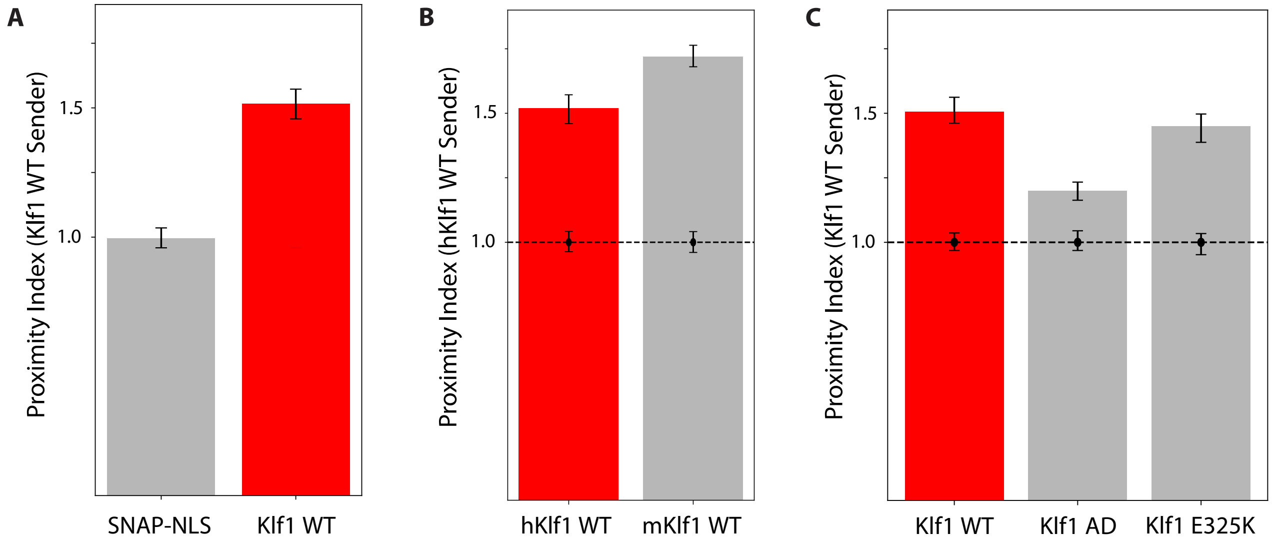

Figure S2. PAPA detects interactions between Klf1 and different Klf1 variants.
(A) PAPA signal between sender-labeled and receiver-labeled wild-type Klf1. **(B)** PAPA signal between sender-labeled human Klf1 and receiver-labeled mouse Klf1. **(C)** PAPA signal between sender-labeled wild-type Klf1 and both receiver-labeled N-terminal Klf1 IDR (AD) and receiver-labeled E325K mutant Klf1.

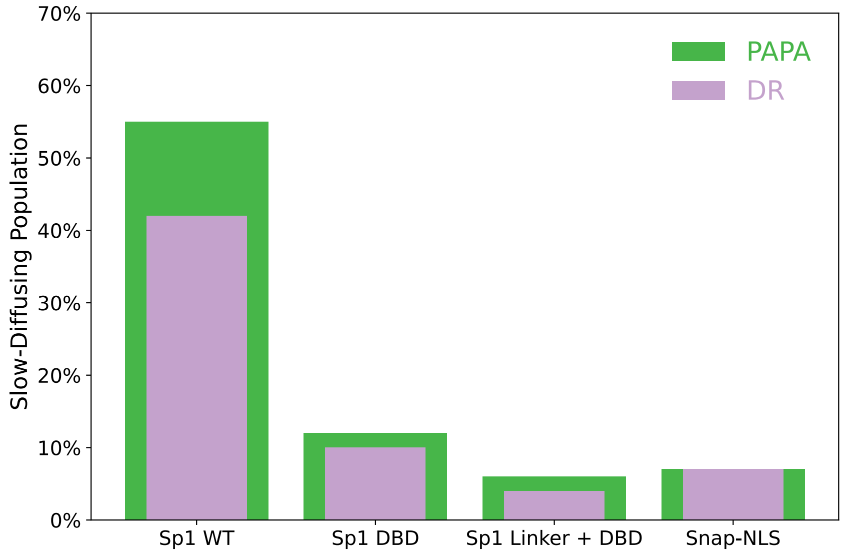

Figure S3. Isolated Sp1 DBD fails to appreciably bind chromatin.
